# Understanding client satisfaction in elderly care: new insights from social resource theory

**DOI:** 10.1007/s10433-020-00591-6

**Published:** 2020-11-21

**Authors:** Ali Kazemi, Petri J. Kajonius

**Affiliations:** Division of Psychology, Education, and Sociology, Department of Social and Behavioural Studies, University West, 461 86 Trollhättan, Sweden

**Keywords:** Social resource theory, Resource theory of social exchange, Person-centred care, Elderly care, Quality of care, Nurse–client interaction, Socioemotional resources

## Abstract

Social resource theory suggests that social interaction can be conceived as resource transaction or exchange with behaviours falling within six fundamental resource categories (i.e. love, status, information, money, goods, and services) organised along two underlying dimensions: particularism–universalism and concreteness–abstractness. With the purpose of extending knowledge about quality of care, this study adopts a novel approach in that it describes and categorises care behaviours using social resource theory instead of using single instances of care behaviour. The categorisation is further used to predict client satisfaction in care services targeting older people. Daily interactions between care staff and older persons were observed in two different residential care facilities using a structured non-participant observation design. The data were analysed using principal component analysis, correlation, and regression analysis. The results confirmed the hypothesis that satisfaction with care services is predicted by resource transactions that are high on the underlying dimensions of particularism and abstractness. Thus, the resource categories of love and status (resource categories high on particularism and abstractness) were shown to be strong predictors of client satisfaction. The use of social resource theory is a novel and appropriate approach to examine person-centred care and satisfaction with care. Also, in addition to addressing potential problems in previous self-report studies on care staff behaviour, the observational technique was highly practical to this service area where dealing with clients not always able to provide feedback directly.

## Introduction

The fragile state of physical health and the associated vulnerability (i.e. needing assistance/care dependency) that comes with high age is an inevitable fact of older residents’ lives in residential care facilities, and this underscores the importance of maintaining and protecting the right of older people to a dignified life and well-being in elderly care (The National Board of Health and Welfare 2012). In fact, the ultimate goal of care is to ameliorate the quality of life in older persons. Mayeroff (1999) asserts that the essence of caring is to initiate a promotive process of well-being and satisfaction in care recipients by focusing on their capabilities. It has also been found that treating the older person with respect and dignity and safeguarding their autonomy are crucial for quality in elderly care (Lothian and Philp 2001; Murphy et al. 2007). This is reminiscent of the basic fact that care is provided in a dyadic context and the quality of the relationship between the caregiver and care recipient is of paramount concern for the outcome of care.

Care has been shown to be composed of two distinct but related aspects which presuppose each other, that is, task and relationship (e.g. Kazemi and Kajonius 2015), which together determines the outcome of care (e.g. well-being of older people, satisfaction with care). In the present study, we go beyond categorising and distinguishing care behaviours in terms of task and relationship and use social resource theory (also called resource theory of social exchange) (Foa 1971; Törnblom and Kazemi 2012a, b) to categorise care behaviours in a more nuanced way. As will be described below, besides helping us to categorise care behaviours social resource theory helps us to describe attributes of various care behaviours as well as to provide a systematic account of how they relate to each other and outcome of care (e.g. client satisfaction).

Residents at residential care homes for older people have been shown to be dissatisfied with the fact that care staff does not take their views into account (i.e. lack of status) (e.g. Berglund 2007). According to Ranheim et al. (2011), this has to do with bureaucratic aspects of care making it more difficult for humanistic aspects of care to manifest itself. As the principles of marketisation have gained ground in elderly care (Dahl et al. 2015), efficiency in terms of low staffing, tight and inflexible schedules but also care routines which make no room for discussions with the clients (cf. Persson and Wästerfors 2009) have increasingly come into focus. This trend has in turn in some cases led to staff performing their caring tasks stressfully which in effect means not always being able to treat the residents as unique individuals (e.g. Fagerberg and Engström 2012).

Treating residents as unique individuals is at the heart of quality in elderly care. Care quality has increasingly come to be equated with the capacity of service providers to satisfy the needs and wants of the service users. Putting the needs, wants, preferences, limitations but also the capabilities of the older person at the centre of care planning and caregiving and providing individualised care services to enhance client satisfaction is referred to as person-centred care (Edvardsson and Innes 2010; McCormack 2004; Slater 2006), also known as user-oriented care (Kajonius and Kazemi 2016a; Kazemi and Kajonius 2015).

Engaging in relational practices (Williams et al. 2009) is crucial for provision of person-centred elderly care (Dewar and Nolan 2013; Liaschenko and Fisher 1999). Williams et al. (2009) argue that in order for staff to engage in relational practices, they must be valued and given recognition as an indispensable part of caring and the staff should receive emotional support to engage in relational practices or what also could be referred to as provision of socio-emotional resources. A too heavy focus on the caring task routines most likely gives rise to a limited resource provision profile which ultimately may adversely affect the older persons’ satisfaction with care.

Attainment of person-centred care is facilitated by various interpersonal and behavioural indicators such as information sharing, providing the clients a say in the care planning process, dignified treatment (Kazemi and Kajonius 2016; Kajonius and Kazemi 2016b, c), asking questions and taking the time to listen to the clients (Coyle and Williams 2001). To date, there is no structured taxonomy of these behaviours and how they relate to satisfaction with care. As the older person is at the centre of attention for the person-centred care approach, measures of care quality tend to include the older person’s satisfaction as the outcome of the care process and services (Stewart 2001). Thus, finding effective ways to capture and understand satisfaction with care is critical to quality care. We believe that viewing elderly care services as resource provision is conducive to gaining a deeper understanding of the care process and improving the perceived quality of care.

In sum, the present study aimed to describe and categorise care behaviours using social resource theory (Törnblom and Kazemi 2012a, b). We also examined to what extent different types of care behaviour categorised according to a taxonomy of resource categories (i.e. love, status, information, goods, and services) predicted satisfaction with care among residents in residential care facilities for older people. Given these aims, the present study contributes to the line of care research that highlights the importance of the nature and quality of interaction in staff–client relationship for achieving client satisfaction and high-quality care (e.g. Eriksson 2002; Fosbinder 1994).

### Social resource theory

**Fig. 1:**
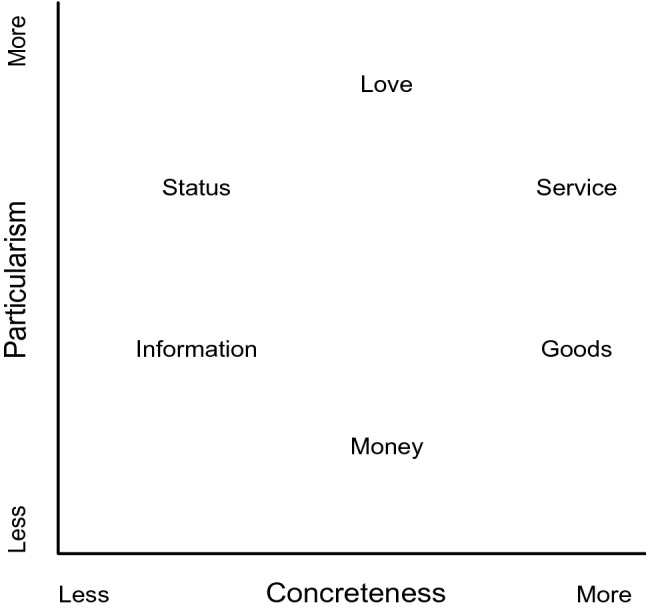
The structure of resources, adapted from Foa (1971)

According to social resource theory, all types of social interaction can be understood in terms of resource exchange. A social resource is defined as “any commodity—material or symbolic—which is transmitted through interpersonal behavior” (Foa and Foa 1974, p. 36; see also Törnblom and Kazemi 2012a, b, p. 34 for definitions and conceptualisations offered by other theorists). Foa (1971) proposed that resources can be decomposed into six distinct resource categories (i.e. love, status, information, services, goods, and money) organised along two dimensions (i.e. universalism–particularism and concreteness–abstractness) (see Fig. 1). This underlying structure is used to determine the functional relationships among resources including what resources people prefer to provide or receive in exchange for what resources.

Foa (1971) defined the six resource categories as follows. Love refers to expressions of affection, liking, warmth, caring, or comfort. Status refers to evaluative judgments conveying prestige, respect, importance, esteem, or other forms of regard. Information entails opinions, advice, instruction, guidance, education, and other forms of enlightenment. Money refers to any coin, currency, voucher, or token with a standard or agreed-upon exchange value. Goods are tangible assets such as material objects, supplies, and equipment. Services refer to any kind of activities performed on one’s body, mind, or possessions and encompass the labour, effort, and energy required to carry out those activities. One should keep in mind that a resource category is a category of the “meaning assigned to actions and not a classification of actions” (Foa and Foa 1974, p. 82). This indicates that different behaviours may be understood and classified as instances of the same resource category depending on the context in which they occur (e.g. the acts of smiling, giving flowers, hugging, affective verbal statements may all convey love).

The axis of particularism–universalism is defined in terms of resource value and reflects the extent to which the value of a specific resource is determined by the identity of the person providing the resource and/or the relationship between the provider and the recipient. Love is the most particularistic resource category, whereas money is the least particularistic (i.e. most universalistic) resource category. Thus, the value of love is contingent on the identity of the provider or the relationship between the provider and the recipient, whereas the value of money is the same regardless of the identity of the provider or the relationship between the provider and the recipient. The other axis (i.e. concreteness–abstractness) refers to the extent to which a resource exists in material form and ranges from completely tangible and concrete to completely intangible and symbolic/abstract. Whereas the transaction or exchange of tangible resources is easily observable (e.g. exchange of money for goods), the use of intangible resources is inferred from overt behaviours of the exchange parties in that specific context (e.g. a hug signals love). Moreover, each resource category encompasses a wide range of concrete instances or subtypes (e.g. examples of information are advice, instruction, threat, etc.).

The *Handbook of Social Resource Theory* (Törnblom and Kazemi 2012a) contains the most up to date presentation of the theory, and interested readers may consult this book for detailed descriptions of basic tenets and various applications of the theory.

### Present study

The objective of the present study was to analyse to what extent various types of care staff behaviours, categorised using social resource theory, account for client satisfaction. Our hypothesis was that in providing care, the more a certain sequence of interaction (i.e. resource transaction) was characterised by staff providing particularistic and abstract resources to the older person, the more satisfied the older person would be. According to social resource theory, high levels of particularism and abstractness would primarily be realised in transactions involving the resource categories of ‘love’ and ‘status’ or meanings assigned to behaviours rooted in these two resource categories.

## Methods

The study was conducted at two different residential care facilities for older persons in a medium-sized municipality in Sweden. These facilities which integrated care and housing provided services to people aged 65 plus around the clock. An older person receives a place after an assessment if their needs exceed a level that makes it impossible to receive home-based care. Thus, characteristic for the residential care facilities was a clientele with multi-functional impairments and serious illness because of high age. Cognitive deficiencies were also common and up to around 50% of the residents had symptoms of dementia. The care facilities had about 30 residents each, with 5–6 daytime staff. The facilities included one-room apartments and a shared dining room and a community hall. The facilities had both indoor and outdoor activities for the residents, and it was possible for the residents to go outside. The clients were all aged 80 plus. The care facilities were chosen based on voluntary response from unit managers in consultation with the department manager in the municipality. The majority of the care staff were women (> 90%, with an approximate mean of 40 years of age).

### Observation instrument

Based on the resource taxonomy provided by social resource theory, non-participant observers rated daily resource transactions from the care staff to the older person. An observation protocol was developed based on the taxonomy proposed by Foa (1971). The overall structure of the observation protocol was organised around five of the resource categories. The category ‘money’ was excluded because transactions and exchanges of money seldom occur in the staff–client relationship in residential care settings in Sweden. Operationalisations of the resource categories are provided in Table 1. The indicators depict behaviours that were noted and rated as described in the Procedure section. Examples of actual behaviours are also given in Table 1.

**Table 1:** Operationalisations of resource categories (predictors) and perceived client satisfaction (outcome variable) based on observations in residential care facilities for older persons

Resource category	Indicators	Example of observed behaviour
Love	Affect	Hugging the older person
	Warmth	Smiling
		Laughing
	Touch	Resting a hand on the shoulder
		Supporting the older person
	Comfort	Asking ‘does it hurt?’, ‘how do you feel?’
Status	Appearance	”You are looking fantastic!”
		”Everyone will be jealous of you”
	Respect	”You’re in charge!”
		”Do you want me to help?”
Information	Advice	”We’ve talked about this—you have to eat!”
	Opinions	”Enough”
	Instructions	”Here is the soup”
		”Please, stand up”
	Announcements	”I have some pills for you”
		”I’ll get you some clean socks”
Money	Not applicable	
Goods	Task objects	Napkin
		Blanket
	Products	Pharmaceuticals
		Bandage
Services	Physical help	Helping the older person out of bed
	Body	Dressing/undressing
	Physical influence	Participating in kitchen activities
	Accessories	Delivering mail
	Time	Spending time with the older person
Satisfaction		
	Overall impression of the interaction	Mood of the client
	Physical, social, affective, and verbal signals from the client	Looking content, expressing verbal appreciation

The observation instrument was piloted on two different occasions for a total of 8 h. These observations were conducted by two research assistants to improve the reliability of observing and recording/classifying care behaviours. In addition, the pilot study served the purpose to introduce and familiarise the care staff and residents to the experience of having research assistants in the care setting. The observers were trained to use the observation protocol but were not aware of the research hypothesis.

### Procedure

Two research assistants conducted structured observations using the observation protocol listing the different types of resources and their concrete instances (Table 1). After a short introduction explaining the purpose of study, the interactions of the day shift care staff and the older residents were observed. The observations started at 07.00 and ended at 17.00 and lasted for four consecutive days. Overall, the reactions from the care staff and their clients to the presence of research assistants and conducting the study were positive. No compensation was given for participation in the study.

A total of *N* = 139 observations was registered to obtain a quantitative measure of resource transactions from the staff to the older person from a third-party observer perspective. An observation was defined as a sequence of interaction between the nursing staff and the older person with a clear start and ending (e.g. guiding and helping the older person to the shared dining room and back to the apartment). Each interaction sequence was rated along the five predefined resource categories (see Table 1). Ratings were done using a Likert scale ranging from 1 (not descriptive at all/never occurred) to 5 (very descriptive/frequently occurred). To illustrate, if the care staff during morning wake-up routines had much body-contact (i.e. frequent touching of a client), this would score 4 or 5 on ‘love’ (i.e. touching was seen as an indicator of the resource category of ‘love’). If the care staff had virtually no physical contact except for helping a client out of bed or to the bathroom, this would score 1 or 2 on ‘love’ for that particular interaction. Example indicators of client satisfaction were smiling, not complaining, giving complements, and being thankful and were scored in the same way as care staff behaviours.

Participation was voluntary and the observational data were made anonymous, with no trackable references to individual care staff or the older persons. Head of the care department as well as the unit managers provided permission to carry out the observations at the residential care facilities. Informed consent was obtained by asking care unit managers and/or the care staff to seek permission on the research assistants’ behalf if the client was not competent to give his or her consent before entering his or her room. The presence of the research assistants was explained to all the care staff and residents, including those who did not participate in the study.

### Statistical analysis

First, we conducted a principal component analysis (PCA) to explore the underlying structure of the data. Kaiser–Meyer–Olkin (KMO) measure of sampling adequacy was checked prior to the PCA. A KMO value of 0.55, exceeding the cut-off point of 0.5, was yielded indicating the adequacy of performing a PCA. We used PCA with an orthogonal rotation (the Varimax method) in extracting the components. An orthogonal rotation, in contrast to an oblique rotation, imposes the restriction that components do not correlate which in this case was deemed appropriate as the axes of particularism and abstractness are treated as independent dimensions in social resource theory (cf. Kline 2002). In determining the number of components to extract we used Kaiser’s criterion (i.e. eigenvalues greater than one) and an examination of a scree plot, a line plot of the eigenvalues of components on the *Y*-axis and the components on the *X*-axis (Tabachnick and Fidell 2001). The extracted components, represented by component scores, were subsequently used in correlation and regression analyses.

Second, we correlated the resource categories and extracted component scores with client satisfaction using Spearman’s correlation coefficient (Field 2013). Following Cohen (1988), a correlation coefficient in the range of 0.10–0.29 was considered to represent a weak association; a correlation coefficient in the range of 0.30–0.49 was considered a moderate association; and a correlation coefficient of 0.50 or larger was considered to represent a strong association (see also Hemphill, 2003). Cut-off point for statistical significance was chosen as *p* < 0.05.

Third, we conducted two linear multiple regression analyses. In the first regression analysis, all resource categories (i.e. love, status, information, goods, and services) were simultaneously entered as predictors of client satisfaction. In the second regression analysis, the extracted components (i.e. particularism and abstractness, and their interaction) were simultaneously entered as predictors of client satisfaction.

All the statistical analyses were conducted in IBM SPSS v.23.

## Results

The PCA provided support for a two-component solution. The two extracted components accounted for 63.6% of the variance. A scree plot also confirmed the adequacy of a two-component solution in support of social resource theory (i.e. particularism and abstractness).

In Table 2, Spearman’s correlations between the resource categories, underlying dimensions of resource categories and client satisfaction, are presented. As predicted, the particularism and abstractness dimensions were both positively related to client satisfaction.

**Table 2:** Spearman correlations between study variables

	1	2	3	4	5	6	7
1. Love	–						
2. Status	0.23**	–					
3. Information	0.30***	0.25**	–				
4. Services	0.30***	− 0.02	0.33***	–			
5. Goods	− 0.37***	− 0.32***	− 0.01	− 0.11	–		
6. Satisfaction	0.39***	0.66***	0.28**	0.11	− 0.23**	–	
7. Particularism	0.56***	0.15	0.75***	0.81***	− 0.04	0.28***	–
8. Abstractness	0.60***	0.75***	0.20*	0.06	0.76***	0.60***	0.22*

^*^p < 0.05; **p < 0.01; ***p < 0.001

In the first regression model, entering the five resource categories (i.e. love, status, information, goods, and services) simultaneously, 49% of the variance in client satisfaction was accounted for (Adj *R*^2^ = 0.49), *F*(5, 133) = 26.98, *p* < 0.001). Only love (*β* = 0.18, SE = 0.06) and status (*β* = 0.63, SE = 0.07) were significant predictors (*p* < 0.001), indicating that these were the resource categories which contributed to the prediction of client satisfaction.

In the second regression model, we used the component scores of the particularism and abstractness dimensions and their interaction to predict client satisfaction. The model accounted for 35% of the variance in client satisfaction (Adj *R*^2^ = 0.35), *F*(3, 133) = 24.31, *p* < 0.001). Both the particularism (*β* = 0.19, SE = 0.06) and abstractness dimensions (*β* = 0.47, SE = 0.07) predicted client satisfaction (*p* < 0.001). The interaction was, however, not statistically significant (*β* = 0.01, SE = 0.06).

## Discussion

The present study viewed the care process in terms of resources provided to the older person by the care staff working in two residential care facilities and investigated perceived satisfaction of the older person following various sequences of interaction differing in terms of resource provision profile. The main finding was that high observer scores on the resource categories of love and status best predicted client satisfaction. Another interesting finding was that the underlying particularism and abstractness dimensions accounted for the relationships between the resource categories and client satisfaction. In other words, manifest behaviours such as acts of respect carry much symbolic value for all clients, and perhaps especially for older persons. Similarly, being treated with kindness and warmth implies a particularistic relationship with the care staff, which was shown to be treasured by the older persons.

We found that provision of love and status (i.e. particularistic resources) were the strongest predictors of client satisfaction. An important practical implication of this finding is that as the value of particularistic resources is contingent on the provider (i.e. the identity of the provider and the provider–client relationship), high levels of turn-over or having different care staff too frequently may create a sense of discontinuity in older persons and thus adversely affect their satisfaction with care. In other words, ‘knowing the person’ requires continuity, and continuity is crucial for provision of high-quality elderly care (e.g. Castle and Engberg 2005; Woodward et al. 2004). Continuity facilitates the exchange of affection and status which were shown to be of utmost importance for satisfaction with care. However, stressed out and frustrated care workers do not provide quality care, and being repeatedly assigned to the most challenging clients can be exhausting and stressful and can lead to depersonalised care (i.e. not treating the older person as a unique individual which is essential to quality care). Thus, although continuity is essential to quality care, it must be balanced with individual workers’ well-being, particularly where individual clients’ care is highly demanding (e.g. challenging behaviours due to dementia). Continuity (and staff retention) in elderly care can be achieved by rostering regular small teams of staff, ensuring consistency for clients and regular relief opportunities for staff (cf. Chenoweth et al. 2010; Mittal et al. 2009). The ultimate aim is to optimise the quality of relationships for both parties to achieve the best possible results in elderly care (e.g. Dewar and Nolan 2013).

What the present study shows in support of previous studies is that provision of socio-emotional resources (i.e. relational practices) is of crucial importance for outcomes of care, in this case, older persons’ satisfaction with care. The “Senses Framework” (Nolan et al. 2006) asserts that high-quality relationships in elderly care include and promote six senses (i.e. security, belonging, continuity, purpose, achievement and significance). This attests the importance of socio-emotional resources (i.e. status and love) for increasing client satisfaction in elderly care. We showed in this paper that care for older people is likely to improve (i.e. higher client satisfaction) when the performed care not only includes performing the routine care tasks but also what may be called extra role care behaviour by giving socio-emotional resources to the older person (cf. Heliker 2009).

Our results confirm previous findings in other respects as well. Status as related to autonomy (e.g. letting the older person decide mundane things and things that they manage to do on their own or with minimal assistance) has been shown to be a crucial feature of well-functioning care relationships (Custers et al. 2011, 2012). Moreover, as observed in the present study, acts of love, which physical contact (e.g. touching hands) is an expression of, has in previous research been shown to be related to positive outcomes in elderly care. For instance, Butts (2001) reported that what she called comfort touch (skin-to-skin touch) and a relational orientation significantly improved self-rated health, self-esteem, and life satisfaction of residents in two residential care facilities.

Kazemi and Kajonius (2015) also showed that relation-orientation was more instrumental in achieving quality care than task-orientation in both home-based care and residential care facilities, suggesting that in performing assisting behaviours, relationship comes first and task comes second for the older person. Care staff may, however, not always regard “relational” aspects of care as part of their job and this may prevent them from engaging in relational practices in their work. Moreover, not seldom their time is limited (Nolan et al. 2001) and the time most often is believed to be enough for performing the must-to-do tasks only. However, what is important to keep in mind is that although engaging in relational practices may be demanding, it does not always need to be time-consuming. Yet, it creates added value for the older person as suggested by the present research findings. In addition, what the staff usually do not consider is what they receive in return. Specifically, the return on investing time in giving socio-emotional resources, such as status and love to the older person, is the sense of meaning and fulfilment that may appear in care workers as they feel that they have succeeded in creating an enriched care environment (Nolan et al. 2006). The exchange of socio-emotional resources promotes what Brown-Wilson (2008) terms “personal and responsive” relationship in which the older resident is recognised as a unique person and the care staff engage in conversations to find out what is important to the resident (i.e. giving them freedom of choice/autonomy and thereby providing them status, which according to our findings, promotes residents’ satisfaction with care). The “personal and responsive” relationship is the contrast to the “pragmatic” relationship characterised by a task-centred focus (i.e. a focus on the care routines rather than on understanding the significance of a particular care routine to the older resident).

The present study was conducted in Sweden. However, there are reasons to believe that its results may apply to elderly care services in other countries. First, social resource theory that we used in the context of the present study to categorise care behaviours was launched as a general and universal theory and has since its formulation received considerable support in different settings and countries (for an overview see Törnblom and Kazemi 2012a), and our results validate the claims of this theory in the context of staff–client interaction in elderly care. Second, the finding that love/affection and status were shown to be the strongest predictors of client satisfaction has parallels in previous research (e.g. Butts 2001; Custers et al. 2012; Dewar and Nolan 2013; Lothian and Philp 2001; Nolan et al. 2002, 2004) discussed earlier. Nevertheless, this does not substitute the need for future studies using social resource theory as a framework to investigate the external validity of the present study’s findings.

### Some reflections upon social resource theory and methodology

Classifying behaviours and assigning them to a resource category is not a straightforward process but depends on the context (Törnblom and Kazemi 2012b). Specifically, although the same behaviour can be assigned to a certain resource category, its meaning may change depending on the motive behind the behaviour, and thus also its resource category affiliation. Similarly, different behaviours may be classified as belonging to the same resource category when they are used to convey the same meaning (e.g. giving someone a flower, smile, or verbal affectionate statements to convey love). Thus, as social resource theory classifies resources in terms of their *meaning* in the very specific context of interaction (Foa 1971), it is crucial to understand whether each party has a similar symbolic interpretation of the transacted resource (e.g. does a hug have the same meaning to both parties?).

Moreover, in the present study, observers focused only on resources that the care staff provided to the older person, and not the other way around. To study the quality of staff–client relationship more comprehensively, future research should focus on the resource *exchange* processes and not one-sided transactions. In this way, by drawing on insights from social resource theory, we can more “fully capture the interdependencies and reciprocities that underpin caring relationships” (Nolan et al. 2002, p. 203) (see also Dewar and Nolan 2013; Nolan et al. 2004 for related discussions on relationship-centred approaches to elderly care).

Furthermore, the dependent variable (i.e. client satisfaction) was limited in that it was rated by the same observers who rated the characteristics of the resource transactions. This may potentially pose a problem to the validity of observed relationships between measured variables and has been discussed in the literature under the rubric of common method biases and common method variance referring to variance that is attributable to the measurement method rather than to the constructs the measures represent (Podsakoff et al. 2003). Thus, confidence in the findings would have been further enhanced if satisfaction had been rated by the older persons themselves as well. However, due to frailty and other aggravating circumstances this was not an option.

Another feature of the present research was that we only looked at positive acts, but staff–client relationships in elderly care settings also entail negative acts which may be initiated from either end of the interaction (e.g. Saveman et al. 1999; Zeller et al. 2009). Aversive interaction in care settings is better understood through the lens of social resource theory. Simply put, positive interactions refer to acts of resource provision, whereas negative interactions refer to withholding or taking away resources (Törnblom and Kazemi 2012b). There are six negative counterparts to the six resource categories, that is, acts signalling disaffection or hate (taking away/withholding love), acts expressing derogation of opinions and abilities or disrespect (taking away/withholding status), acts of misrepresentation of facts and deceit (taking away/withholding information), acts of stealing (taking away/withholding money), acts of damaging properties and belongings (taking away/withholding goods), and acts causing pain or any other discomfort to body (taking away/withholding services). Thus, social resource theory also helps to explain the nature of dysfunctional dyadic relationships between staff and their clients in care settings and how and why different types of “taking away” behaviours may affect the outcome of care (e.g. satisfaction/dissatisfaction with care).

An important methodological feature of the present study worthy of notice was that in contrast to previous research in which care workers provide self-reports on the way they work towards their clients (e.g. White et al. 2008; De Witte et al. 2006), we conducted non-participant observations, using third-party observers trained to observe and categorise behaviours without being aware of the research hypothesis. By conducting observations, we believe that we have tackled common potential problems in self-report studies (i.e. self-serving biases and social desirability effects in reporting on how you behave towards the older person) (Paulhus 1986). In addition, the Hawthorne effect (also known as the observer effect referring to the phenomenon where workers modify aspects of their behaviour towards being more socially acceptable as a reaction to the presence of an observer, cf. Monahan and Fisher 2010) is probably not an issue since observations were taken over 4 days and this effect can be overcome by long periods of observation where the staff become accustomed to the presence of the observer. Moreover, the likelihood of observer effects decreases when using double blind procedures, that is, both the observers and the observees are unaware of the purpose of the study (e.g. Kirk 2012).

## Concluding remarks

How staff behaves towards their clients is fundamental to achievement of high-quality care. The present research extends our knowledge about how care behaviours may be grouped and provides an explanation as to why certain care behaviours may be more important to client satisfaction than others. Thus, we believe that the use of social resource theory is a novel and highly appropriate approach to examine person-centred care and satisfaction with care. Also, the observational technique is highly practical to this service area where dealing with clients not always able to provide feedback directly. In conclusion, we hope that this study contributes to practice and studies of client care and workforce development in the elderly care sector.
